# Does Spouse’s Dementia Diagnosis Make Individuals Skimp on Health Care?

**DOI:** 10.1093/geroni/igaa057.1537

**Published:** 2020-12-16

**Authors:** Yi Chen

**Affiliations:** University of Southern California, Irvine, California, United States

## Abstract

Dementia is a costly disease that places great burden on individuals, families, and health care systems. The substantial time and financial resources taken away by living with persons with dementia (PWDs) may make their spouses forgo needed health care, thus deteriorating long-term health. To quantify the effect of dementia on spouses’ health investment, I employed a difference-in-difference approach, comparing use of preventive services and doctor visits before and after spouses’ dementia onset. Using Health and Retirement Study (HRS) with linkage to Medicare claims, I identified 650 older adults whose spouses had incident dementia during 1993 to 2007, and matched them to 1,816 controls whose spouses were dementia-free. Primary analysis reveals that individuals whose spouse had dementia did not change their use of most health services, relative to dementia-free controls. In stratified analysis, middle-class individuals skimped on flu shot and diabetes screening. Providing help for activities of daily living (ADLs) was associated with 1.9 less doctor visits, the effect of which was stronger among females (2.5 less visits). Help with instrumental activities of daily living (IADLs) was not a predictor of any utilization outcome. In conclusion, externalities of dementia imposed on family members are more profound and complex than deprivation of time. Certain subgroups were worse off in health investment when facing the trade-off between caring for spouses with dementia and caring for themselves. When understanding dementia burden, the externality imposed on spouses and its heterogeneity should be considered.

